# Advanced ultrasound diagnosis of extrahepatic bile duct lesions

**DOI:** 10.1007/s10396-024-01491-3

**Published:** 2024-10-21

**Authors:** Shinji Okaniwa

**Affiliations:** Department of Gastroenterology, Iida Municipal Hospital, 438 Yawata-Machi, Iida City, Nagano 395-8502 Japan

**Keywords:** Bile duct, Differential diagnosis, Ultrasound, Endoscopic ultrasound, Intraductal ultrasound

## Abstract

Ultrasound (US) has high specificity and sensitivity, and it should be performed first for patients with suspicion of biliary tract cancer. However, the complicated anatomy in addition to the gas images makes it difficult to delineate the entire extrahepatic bile duct (EHBD). The keys to depiction of EHBD are the "J" shape manipulation in the left lateral decubitus position and the use of magnified images with high-frequency transducers. Furthermore, indirect findings such as gallbladder (GB) distension, BD dilatation, and debris echo in the GB and BD are also important for detecting occult lesions, particularly in the ampullary region of Vater. For the differential diagnosis of BD wall thickening, the spreading pattern in the long and short axial directions should be assessed first. Then, the characteristics of the innermost hyperechoic layer (IHL) and outermost hyperechoic layer (OHL) should be evaluated. Asymmetrical wall thickening, absence of IHL, and presence of irregularity or discontinuity in OHL are characteristic patterns of cholangiocarcinoma (CCA). Because CCA is the most common BD polypoid lesion, it is important to diagnose tumor extension and depth invasion in addition to differential diagnosis. Nodular-type CCA is usually hypoechoic and more likely to invade vertically. In contrast, papillary-type CCA is often hyperechoic and extends laterally. Contrast‑enhanced US may be useful for evaluating these findings. However, if the possibility of CCA cannot be ruled out or a definitive diagnosis is needed, a transpapillary biopsy or endoscopic US-guided tissue acquisition should be considered.

## Introduction

Extrahepatic biliary tract tumors arise from the perihilar bile duct (BD) to the ampulla of Vater and are classified as BD, gallbladder (GB), and ampullary tumors. Although diagnostic imaging advances have made it possible to diagnose these tumors at an earlier stage than in the past, most patients still have advanced cancer at diagnosis, and many of them are unresectable.

Since ultrasound (US) is a simple and noninvasive modality without radiation exposure, it is widely used for cancer screening and health checkups [1, 2]. According to the biliary tract cancer clinical guidelines [3], US, which has high specificity and sensitivity, should be performed as the first step for patients with suspicion of biliary tract cancer. In Thailand, it has been reported that successive US every 6 months can detect early-stage cholangiocarcinoma (CCA) and premalignant lesions and improve the prognosis of CCA [4].

However, the complicated anatomy in addition to the gas images of the gastrointestinal tract makes it difficult to detect lesions in the extrahepatic bile duct (EHBD) and papilla of Vater. Although US can detects tumors in more than 70% of patients with BD cancer [5, 6] and GB cancer [7], US only detects less than 30% of ampullary cancer [8]. Sometimes indirect findings such as GB distension, BD dilatation, and debris echo in the GB and BD are important clues for detecting occult neoplastic lesions of the BD and ampulla of Vater [1].

This review highlights how to visualize the entire EHBD using US first, and then addresses indirect findings. Finally, the differential diagnosis of BD and ampullary lesions, including findings based on endoscopic ultrasound (EUS) and intraductal ultrasound (IDUS), is discussed.

## How to visualize the entire EHBD (Fig. 1)

The EHBD is classified into the perihilar BD and distal BD. The perihilar BD is defined as the area from the right and left hepatic ducts to the confluence of the cystic duct, and the distal BD is defined as the area from the confluence of the cystic duct to its penetration into the duodenal wall. The ampulla of Vater is the area from the penetration of the distal BD into the duodenal wall to the papillary orifice, which is surrounded by the Oddi's muscle. The EHBD runs anterior to the long axis of the portal vein (PV) from the porta hepatis to just above the superior border of the pancreas, and then departs from the PV in the right lateral direction through the pancreatic head ending in the ampulla of Vater.

**Fig. 1:**
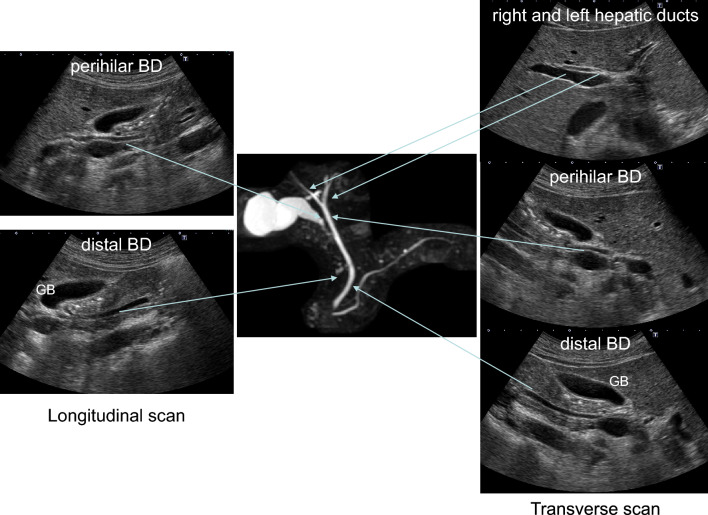
Typical US images of EHBD. *GB* gallbladder

There are three pitfalls for the EHBD: the left and right hepatic ducts, the distal BD adjacent to the duodenum, and the ampulla of Vater. For the left and right hepatic ducts, evaluation just above the horizontal segment of the PV in the upper abdominal transverse scan is most important. Although most sonographers use the supine position to visualize the EHBD, the left lateral decubitus position moves the pancreatic head toward the ventral side and straightens the EHBD, making it easier to delineate the entire distal BD in most cases. However, the ampulla of Vater is still challenging to delineate. As mentioned above, the left lateral decubitus position usually facilitates delineation of the distal BD duct near the duodenum, and the right lateral decubitus position after liquid-filled stomach methods [9, 10], in which fluid accumulates in the duodenal lumen, may be useful in some cases.

To detect BD lesions earlier, the following four US findings mentioned in the manual for abdominal ultrasound in cancer screening and health checkups lists are useful: morphological abnormalities, wall thickening, protruded or mass lesion (polyp), and other findings (stone image, pneumobilia and debris echo) [1]. Similar to the GB, detailed evaluation with magnified images using high-frequency convex or linear transducers is essential to evaluate these US findings. Furthermore, simultaneously examining the pancreatic head region while observing the distal BD may help to detect pancreatic carcinoma. In this review, three representative methods of BD scanning are discussed in the following sections.

### Right subcostal longitudinal scan in the left lateral decubitus position

**Fig. 2:**
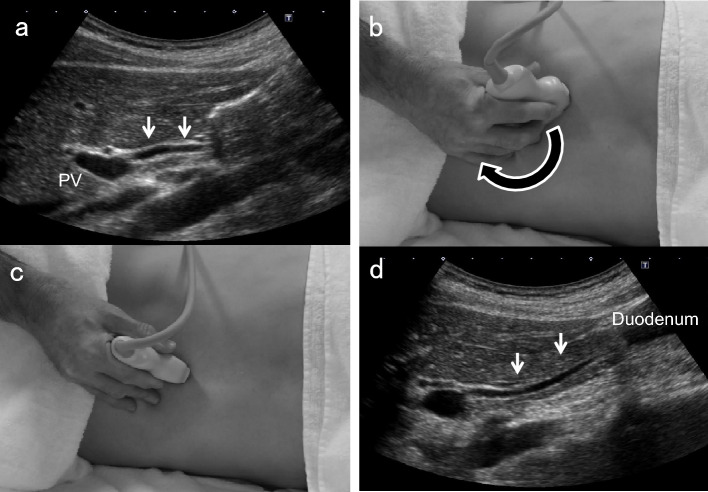
“J" shape manipulation in right subcostal longitudinal scan. After identifying the perihilar BD (↓) anterior to the PV (**a**), rotate the probe clockwise (**b**, **c**), and advance it toward the patient's right side to the foot ("J" shape manipulation). The distal BD near the duodenum (↓) can be delineated (**d**). (Modified reprint from Ref. [10])

**Fig. 3:**
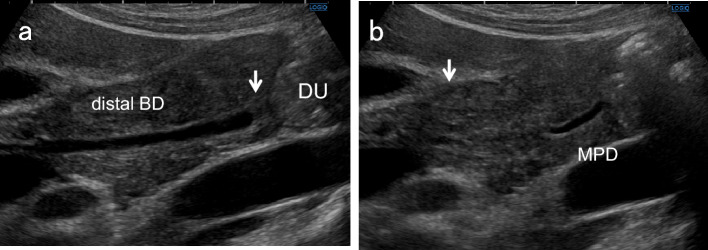
US images of the distal BD and main pancreatic duct adjacent to the duodenum. A detailed evaluation with magnified images using high-frequency convex transducers can depict the papillary fold (↓) inside the distal BD (**a**) and main pancreatic duct (**b**) adjacent to the duodenum. *DU* duodenum, *MPD* main pancreatic duct

Place patients in the left lateral decubitus position and instruct them to hold their breath at a light inspiratory level. After defining the long-axis image of the PV running dorsal to the neck of the GB, delineate the perihilar BD running just anterior to the PV. Move the probe gradually toward the foot, pushing away the gastrointestinal gas with the probe. During this maneuver, the probe should not be pressed hard but rather softly. Even when patients are not holding their breath, the probe should be gently pressed against the body surface to keep the gas away. By rotating the probe clockwise in a "J” shape and advancing to the foot, the distal BD near the duodenum can be visualized. Once the entire course of the EHBD has been delineated, relaxing the pressure of the probe as much as possible often produces good images of the EHBD with a clear lumen. The key point of the depiction of EHBD in this scan is the "J" shape manipulation (Fig. 2) [11]. Detailed evaluation with magnified images using high-frequency transducers can depict the distal BD and the main pancreatic duct adjacent to the duodenum (Fig. 3). In succession, observe the pancreatic head around the distal BD.

### Right subcostal transverse scan in the left lateral decubitus position

**Fig. 4:**
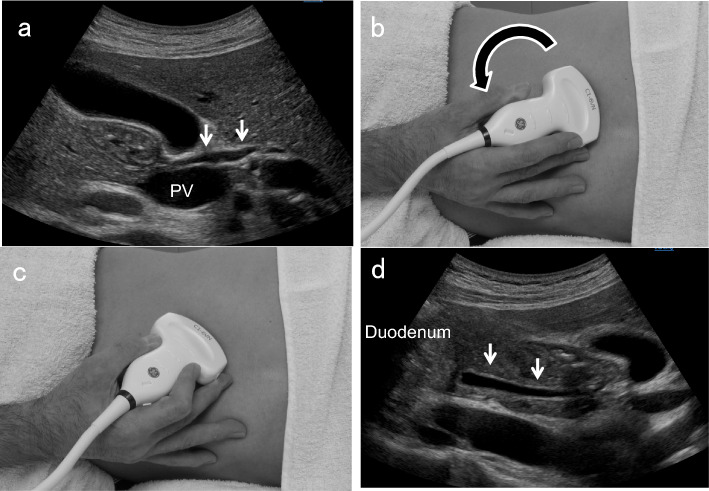
Counterclockwise rotation in right subcostal transverse scan. After identifying the perihilar BD (↓) posterior to the GB neck (**a**, **b**), rotating the probe counterclockwise while demonstrating the perihilar BD (**c**) allows visualization of the distal BD (↓) (**d**)

After identifying the horizontal segment of the PV, define the right and left hepatic ducts located just anterior to it. Next, demonstrate the GB neck on the right side of the right hepatic duct and delineate the perihilar BD posterior to it. Thereafter, rotating the probe counterclockwise gradually and advancing to the foot while demonstrating the perihilar BD enables visualization of the distal BD (Fig. 4). In this scan, note that the ampulla of Vater is on the left side of the screen. In succession, observe the pancreatic head around the distal BD.

### Upper abdominal transverse scan in the supine position

**Fig. 5:**
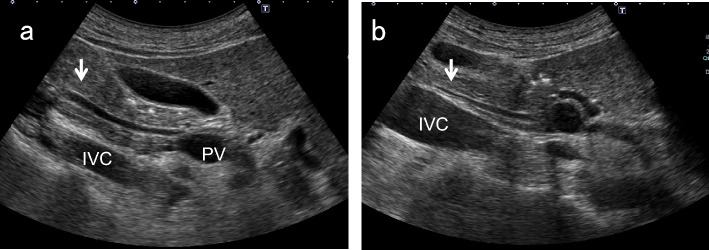
Comparison of distal BD images in the left lateral decubitus position and in the supine position. The distal BD image near the duodenum in the left lateral decubitus position (**a**) tends to have a clearer lumen than in the supine position (**b**). *IVC* inferior vena cava

After identifying the pancreatic body in the upper abdominal transverse scan, move the probe to the right until the image of the pancreatic head is detected. After defining the short-axis images of the EHBD, rotate the probe counterclockwise gradually to delineate the long-axis images of the distal BD, running between the ventral and dorsal pancreas to the duodenum. The distal BD image near the duodenum in the left lateral decubitus position tends to have a clearer lumen than that in the supine position (Fig. 5).

## Indirect findings of occult bile duct and ampullary lesions

As mentioned above, the distal BD adjacent to the duodenum and the ampulla of Vater are prone to be pitfalls. Therefore, indirect findings are especially useful for detecting occult tumors in these regions. In addition to US findings of the EHBD, we also describe those of the GB (distension and debris echo) here.

### GB distension (Fig. 6)

The Courvoisier sign [12] indicates the presence of an enlarged GB that is non-tender and accompanied by jaundice. The etiology is unlikely to be gallstones and more likely to be a malignant pancreaticobiliary mass lesion obstructing the distal BD.

**Fig. 6:**
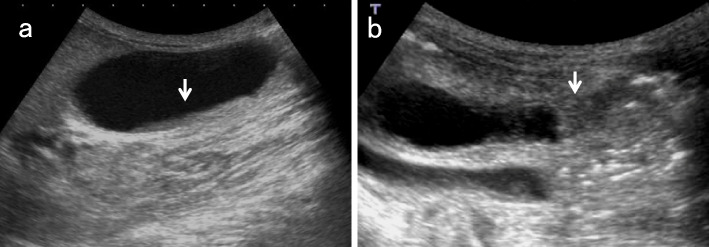
Ampullary carcinoma diagnosed based on debris echoes in the distended GB. The right subcostal longitudinal scan in the supine position shows debris echoes (↓) in the distended GB (a). An ampullary carcinoma (↓) is delineated in the left lateral decubitus position (b)

Although the study was based on magnetic resonance cholangiopancreatography (MRCP), there was a significant difference in GB volume between the group with biliary obstruction due to cholelithiasis and the group with biliary obstruction due to neoplasm or stricture [13]. A pathological study also suggested that chronically increased ductal pressure is the probable cause of dilated GB seen in malignant obstruction of the BD [14].

In the manual for abdominal ultrasound in cancer screening and health checkups [1], GB distension is defined as a maximum short diameter of 36 mm or larger, and it is recommended to evaluate the distal BD up to the duodenum.

### EHBD dilatation (Fig. 7)

EHBD dilatation is also a useful indirect finding of biliary congestion. Although the Japanese clinical practice guidelines for congenital biliary dilatation [15] recommend estimating the inner diameter of the most dilated site of the common BD as the maximum diameter, the manual for abdominal ultrasound in cancer screening and health checkups [1] recommends measuring from the beginning of the anterior wall echo to the beginning of the posterior wall echo, and defines EHBD dilatation as 8 mm or more. Regarding post-cholecystectomy patients, a South Korean prospective study [16] showed that asymptomatic BD dilatation of up to 10 mm was physiological, and that dilatation of 11 mm and more should be considered pathological abnormal dilation.

**Fig. 7:**
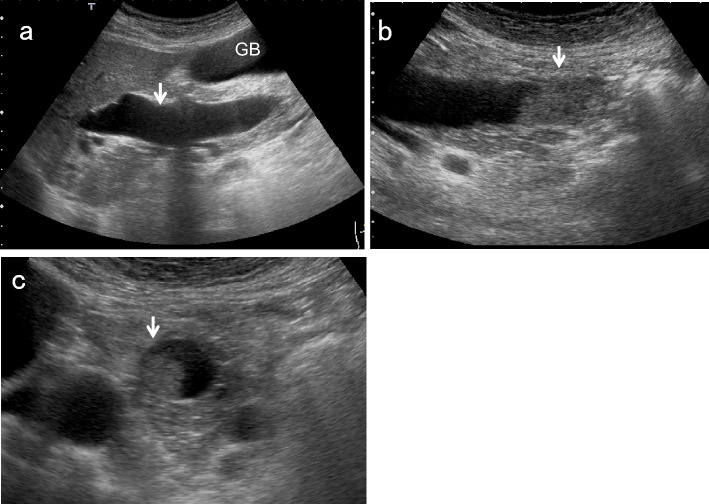
Ampullary carcinoma diagnosed based on BD dilatation. The right subcostal longitudinal scan shows the dilated EHBD (↓) (a). A high-frequency convex transducer delineates an ampullary carcinoma (↓) (b). The layer structure of the wall beneath the tumor (↓) is preserved in the magnified short-axis image (c). *GB* gallbladder

According to a systematic review of patients with incidentally identified dilated BD [17], the most commonly identified causes were choledocholithiasis and chronic pancreatitis. However, malignancies including pancreatic carcinoma, CCA, and ampullary carcinoma were also identified in 12%. In addition, note that early intraductal papillary neoplasms of bile duct (IPNB) sometimes only show BD dilatation without intraductal mass lesions even when contrast-enhanced ultrasound (CEUS) is performed [18, 19].

Potential predictors of malignancy include jaundice, age, and coexistent intrahepatic duct dilatation, and the diameter of the EHBD has no relationship [17]. Therefore, the decision to perform additional detailed examination for incidentally discovered BD dilatations (≥ 8 mm or ≥ 11 mm after cholecystectomy) should be made carefully with reference to clinical symptoms and blood tests.

**Fig. 8:**
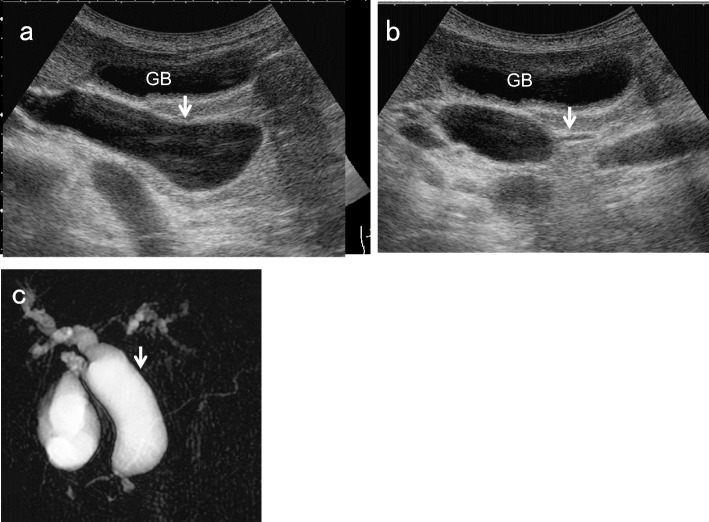
Congenital biliary dilatation (CBD). The EHBD (↓) is dilated cystically (↓) (**a**) and steeply narrowed within the pancreas (**b**) (↓: common channel). MRCP shows CBD with pancreaticobiliary maljunction (PBM) (**c**). (↓: dilated EHBD). *GB* gallbladder

For EHBD dilatation, the shape of the dilatation is also important. In cases with cystic or fusiform dilatation, it is necessary to consider congenital biliary dilatation (CBD) [1, 15] (Fig. 8). Because most CBD cases are associated with pancreaticobiliary maljunction (PBM), which is a risk factor for GB and BD cancer, a detailed examination should be performed even in the absence of mass lesions or other abnormalities [1].

### Debris echo (Fig. 6)

Debris echoes are small, point-like echoes that are suspended or deposited in the GB and BD and are mobile with positional changes. Pathologically, they reflect inflammatory products such as fibrin, necrotic material, pus, and concentrated bile that have accumulated in the GB.

The main cause is inflammatory changes of the GB, such as acute cholecystitis, but bile stasis due to distal BD obstruction and prolonged fasting are also potentially responsible. Although cholecystitis is often associated with diffuse wall thickening, bile stasis due to BD obstruction is not associated with wall thickening. Furthermore, the sonographic Murphy’s sign, which is specific for acute cholecystitis, is usually negative in bile stasis. Therefore, debris echoes in GB without wall thickening or Murphy’s sign should be considered for occult BD and ampullary lesions.

## Morphological classification of US appearance

**Fig. 9:**
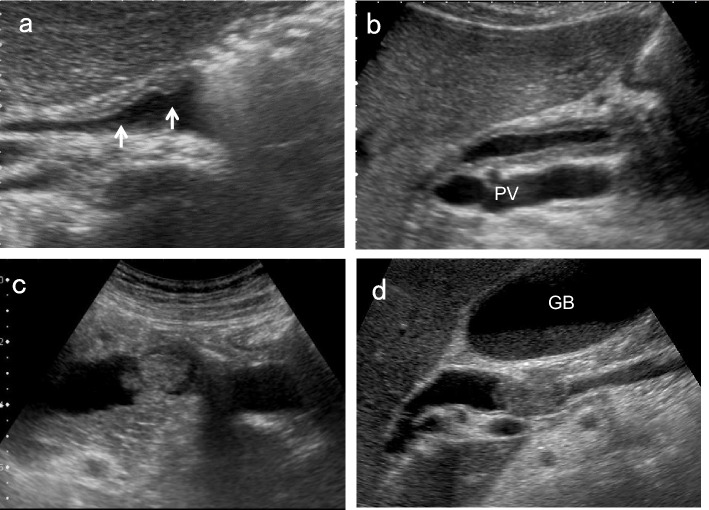
Morphological classification of US appearance. The US appearance of BD lesions is broadly divided into wall thickening (BDWT) (**a** localized type ↑, **b** diffuse type) and polypoid lesions (BDPLs) (**c** papillary-type, **d** nodular-type)

The US appearance of BD lesions is broadly divided into wall thickening (BDWT) and polypoid lesions (BDPLs) (Fig. 9). This classification is also used in US cancer screening [1] and is useful for differential diagnosis.

BDWT is divided into diffuse BD wall thickening with a maximum wall thickness of 3 mm or more (diffuse BDWT) and localized presence of an internal hypoechoic layer regardless of wall thickness (localized BDWT) [1]. Magnified observation with a high-frequency transducer is strongly recommended for detecting BDWT, particularly localized BDWT. Short-axis images, either concentric or eccentric, are most important for differential diagnosis in BDWT.

BDPLs are defined as focal elevation or protrusions that are distinguishable from the surrounding mucosa and can be classified into the papillary or nodular type. Because there are few benign diseases in the EHBD, such as cholesterol polyps of the GB, the diagnosis of tumor spreading and depth invasion are also important. With regard to tumor spreading, the incidence of superficial extension, defined as noninvasive cancerous extension of 20 mm or more, is significantly higher for the papillary type (46.5%) than for the nodular type (12.2%) (p < 0.0001) [20]. For the diagnosis of depth invasion, magnified short-axis observation with a high-frequency transducer is strongly recommended (Fig. 7).

## Differential diagnosis of BDWT

The normal BD wall is composed of four layers: mucosa, fibromuscular layer, subserosa, and serosa. Under favorable conditions and special diseases, US, especially when employing high-frequency transducers, can identify two or three layers: an inner hypoechoic layer and an outermost hyperechoic layer (OHL), or an innermost hyperechoic layer (IHL), a middle hypoechoic layer, and an OHL. According to a comparative study of IDUS images and histological structures of the BD wall, the inner hypoechoic layer contains not only the mucosa and fibromuscular layer but also the fibrous layer of the subserosa [21]. Because studies in GB have reported that the source of IHL is mostly interface echo [22] and also includes the mucosa [23], the BD wall is thought to be similar.

Because BDWT reflects inflammatory changes or neoplastic lesions of the BD, the differential diagnosis should include flat-type CCA, IPNB, cholangitis, primary sclerosing cholangitis (PSC), IgG4-related sclerosing cholangitis (IgG4-SC), and biliary debris, etc. Because reports of US for BDWT are still limited, imaging features obtained from other modalities such as EUS, IDUS, and CT are also cited.

### Long- and short-axis spreading pattern

Inflammatory changes of the BD are a reflection of systemic disease and are considered to have a broader and homogenous impact than neoplastic diseases. Although the study was based on CT images [24], diffuse and concentric thickening is usually observed in patients with inflammatory diseases such as acute cholangitis. IgG4-SC usually spreads extensively along the biliary tract, showing diffuse wall thickening [25], and IDUS depicts symmetrical circular thickening with smooth inner and outer margins [26, 27]. According to a study based on IDUS, symmetrical wall thickening of the BD is detected significantly more often among IgG4-SC patients (100%) than among PSC patients (20%) or CCA patients (8.3%) (p < 0.05) [26]. Another study also showed that symmetrical wall thickness was significantly more common in IgG4-SC patients (73%) than in CCA patients (18%) (p < 0.01) [27].

**Fig. 10:**
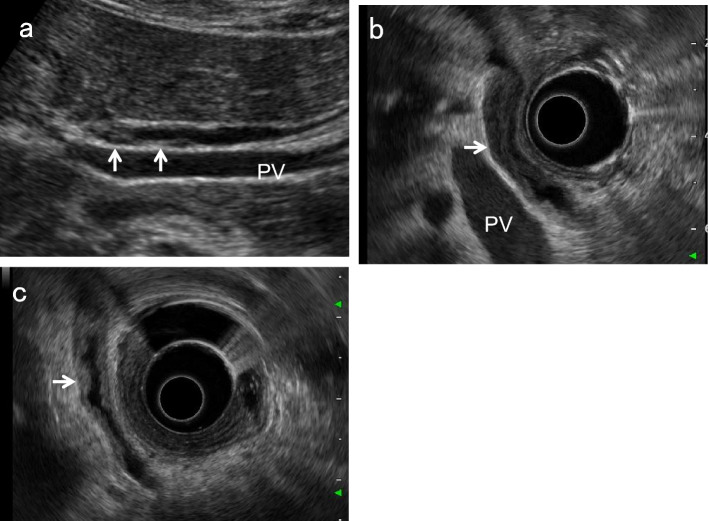
IPNB with extensive wall thickening. A high-frequency transducer shows slightly elevated irregular hypoechoic wall thickening in the right hepatic duct (**a**) (↑: IPNB). EUS shows diffuse extension of IPNB from the perihilar BD to the distal BD (**b**) (↑: IPNB). *PV* portal vein

On the other hand, localized and eccentric thickening of the BD wall is the predominant pattern with neoplastic lesions. However, IPNB, which is a rare variant of BD tumor that exhibits a spectrum from benign to malignant [28], sometimes spreads widely along the intra- and extrahepatic BD and GB, showing diffuse wall thickening (Fig. 10). Liu et al. reported that the mean lengths of measurable intraductal papillary adenomas and papillary adenocarcinomas were 2.5 ± 11 mm (range 12–42 mm) and 56 ± 20 mm (range 33–98 mm), respectively (p = 0.004) [18]. Furthermore, PSC, which is a progressive biliary disease, also presents with eccentric wall thickening [24, 26].

**Fig. 11:**
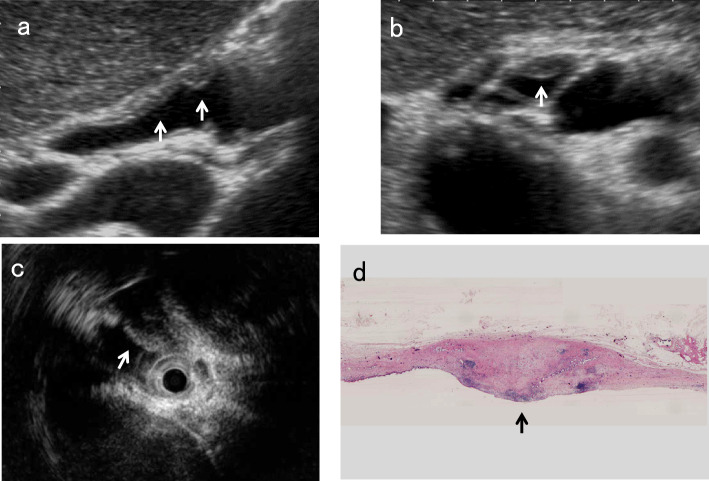
Long and short axial images of an early cholangiocarcinoma (CCA). A high-frequency transducer shows focal irregular wall thickening without IHL (↑) on the long-axis image (**a**) and asymmetrical eccentric wall thickening (↑) on the short-axis image (**b**). IDUS also shows eccentric hypoechoic wall thickening (↓) (**c**). Macroscopic finding of resected tissue at the same site (↑) (**d**)

Because short-axis images obtained using high-frequency transducers are similar to IDUS images (Fig. 11), evaluating the shape of BD wall thickening on short-axis images using high-frequency transducers may be useful in differentiating inflammatory changes from PSC or CCA.

### Layer structure

#### Characteristics of innermost hyperechoic layer (IHL)

**Fig. 12:**
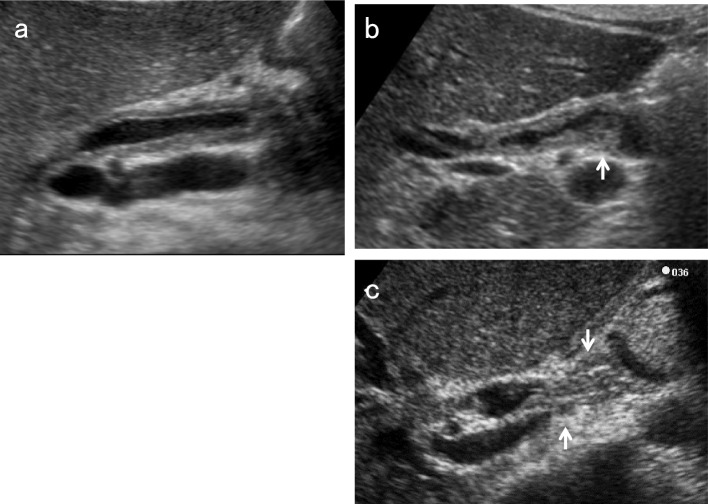
US images of the typical layer structure of wall thickening. An AIP case shows typical three-layered thickening with IHL (**a**). IPNB shows an irregular surface without IHL and irregularity of the OHL (↑) (**b**). PSC shows wall thickening with an indistinct lumen and projection of the OHL to the outside (↓/↑) (**c**)

According to studies in the GB, the source of IHL is mostly interface echo [22], and also includes the mucosa [23]. Because inflammatory thickening of the BD wall is associated with congestion and edema, the mucosal surface is well defined unless accompanied by epithelial erosion or ulceration. Wall thickening of IgG4-SC is caused by marked transmural lymphoplasmacytic infiltration and fibrosis [25, 29], and unlike PSC, the epithelium shows no cell damage or inflammatory cell infiltration. On the other hand, CCA arises from the BD epithelium and causes mucosal irregularity or disruption in most cases. These differences in the mucosal surface affect the characteristics of IHL. This means that IHL is more likely to be seen in inflammatory wall thickening, including IgG4-SC, whereas loss or discontinuity of IHL is more likely to occur in CCA and PSC (Figs. 9, 12).

IHL thickening is also useful for differentiation. According to a study based on GBCs [23], when low papillary tumors aggregate on the mucosal surface, distortion of mucosal structures and echo scattering may occur, causing IHL thickening.

#### Three-layered thickening (high-low–high pattern)

According to the clinical diagnostic criteria of IgG4-SC 2012 [30], diffuse or segmental narrowing of the intrahepatic and/or extrahepatic BDs associated with a thickened BD wall is included as a characteristic biliary finding. Because IgG4-SC does not show any cell damage or inflammatory cell infiltration in the epithelium, a well-defined mucosal surface results in a distinct IHL (interface echoes). Furthermore, diffuse lymphoplasmacytic infiltration and marked interstitial fibrosis reflect a marked thickened middle hypoechoic layer showing the characteristic three-layered (high-low–high pattern) wall thickening [27] (Fig. 12). On the other hand, PSC showed disappearance of the three-layer structure in all cases in an IDUS study [31].

#### Irregularity or disruption of outermost hyperechoic layer (OHL)

Flat-type CCA usually presents with BD stricture, and most biliary strictures are due to malignant diseases, including CCA (76–85%) [32]. However, up to 24% of perihilar BD strictures are benign lesions, including IgG4-RD, PSC, eosinophilic cholangitis, and response to infection or ischemia [33].

Since tumor invasion into the adipose layer of the subserosa causes irregularity of the OHL and tumor invasion beyond the subserosa causes disruption of the OHL, both irregularity and discontinuity of the OHL suggest malignant wall thickening (Fig. 12). According to a study using IDUS, an intact outer margin of stenotic area in IgG4-RD, PSC, CCA was seen at a rate of 100%, 10%, and 8.3%, respectively, and it was suggested to be a characteristic IDUS feature in IgG4-SC [26].

Using the criteria for a malignant stricture (disruption of the trilaminar BD wall, a hypoechoic mass lesion larger than 5 mm, or a wall thickness of more than 3 mm with an irregular outer edge of the BD), EUS has a sensitivity of 79–93% and a specificity of 94–97% in diagnosing malignant biliary stricture [34]. Furthermore, Naitoh et al. reported that diverticulum-like outpouching in the OHL on IDUS was observed in 67% of PSC, and was a specific IDUS finding for PSC [31].

#### Maximum wall thickness in non-stricture area

According to a study using IDUS, the wall thicknesses of the stenotic and dilated areas in IgG4-RD, PSC, CCA were 3.7 ± 0.9 mm, 2.6 ± 0.9 mm, and 2.8 ± 0.6 mm, respectively, and 1.9 ± 0.5 mm, 0.8 ± 0.4 mm, and 0.9 ± 0.5 mm, respectively [26]. Naitoh et al. reported that the mean wall thickness of proximal non-stricture BD was 1.2 ± 0.3 mm in IgG4-SC and 0.5 ± 0.1 mm in CCA, with the wall thickness in IgG4-SC being significantly greater than that in CCA (p < 0.0001) [27]. They also concluded that a BD wall thicker than 0.8 mm in the non-stricture area may be suggestive of IgG4-SC [27].

### Contrast-enhanced US (CEUS)

Although the study was conducted using EUS and IDUS, Hyodo et al. [35] reported that the thickened BD wall in IgG4-RD was strongly enhanced 30 s after administration of Levovist and reached a peak at 120 s. They also reported that contrast-enhanced EUS showed reduced enhancement of the BD wall and attenuation of BD thickening after initiation of steroid therapy. Furthermore, CEUS facilitates evaluation of the inner layer of the BD, making it easier to determine that the inner surface of the BD is smooth in IgG4-SC and irregular in PSC [36].

From the above, the spreading pattern in the long and short axial directions should be assessed first in the differential diagnosis of BDWT. In particular, focal and eccentric thickening of the BD wall is the characteristic pattern of CCA. Thereafter, the characteristics of the IHL and OHL should be evaluated. Although PSC must be excluded, the absence of IHL or the presence of irregularity or discontinuity in the OHL is the characteristic pattern of CCA (Table 1). CEUS may be useful to evaluate these findings. However, if CCA cannot be ruled out or a definitive diagnosis is necessary, transpapillary BD biopsy [27] and EUS-guided tissue acquisition should be considered [37].

**Table 1:** Characteristic US images of important BDWTs

	IgG4-SC	PSC	IPNB	CCA
Spreading pattern in long axial directions	Diffuse > > localized	Diffuse > localized	Diffuse > localized	Localized > diffuse
Spreading pattern in short axial directions	Symmetrical, concentric thickening	Asymmetrical, eccentric thickening	Asymmetrical, eccentric thickening	Asymmetrical, eccentric thickening
IHL	Well recognized	Loss or discontinuity	Loss or discontinuity	Loss or discontinuity
Outermost hyperechoic layer	Regular and well maintained	Projection of the outermost hypoechoic layer to the outside	Irregular or disruption in advanced cases	Irregular or disruption in advanced cases

*IgG4-SC* immunoglobulin G4-sclerosing cholangitis, *PSC* primary sclerosing cholangitis, *IPNB* intraductal papillary neoplasm of the bile duct, *CCA* cholangiocarcinoma, *IHL* innermost hyperechoic layer

## Differential and tumor extension diagnosis of bile duct polypoid lesions (BDPLs)

Unlike GB tumors, the proportion of benign tumors in EHBD accounts for only 6% [38], and CCA, IPNB, neuroendocrine neoplasm (NEN), amputating neuroma (AN), and biliary sludge, etc., should be differentiated. Furthermore, diagnosis of lateral spreading and depth invasion are also important.

As described in the morphological classification, BDPLs can be broadly classified into papillary and nodular types (Fig. 9). According to a clinicopathological study of CCA, the incidence of superficial spreading, which is continuous with the main tumor and extends ≥ 20 mm into the mucosal epithelium, was 25.0% in distal CCA and 11.1% in perihilar CCA (p < 0.0001) [20]. Furthermore, the frequency of superficial spreading differs greatly depending on the gross type, and its incidence was 46.5% in the papillary type and 12.2% in the nodular type (p < 0.0001) [20]. Therefore, classification of BDPLs into papillary and nodular types is useful not only for the differential diagnosis but also for the diagnosis of tumor extension (Table 2).

**Table 2:** Characteristics of papillary-type and nodular-type CCA

	Papillary-type (including IPNB)	Nodular-type
Common site	Distal BD	Perihilar BD
Echo texture	Hyperechoic	Hypoechoic
Type of tumor extension	Superficial lateral spreading	Vertical invasion
Dilation of the proximal bile duct	−~+	+~ ++

*IPNB* intraductal papillary neoplasm of the bile duct

### Echogenicity

**Fig. 13:**
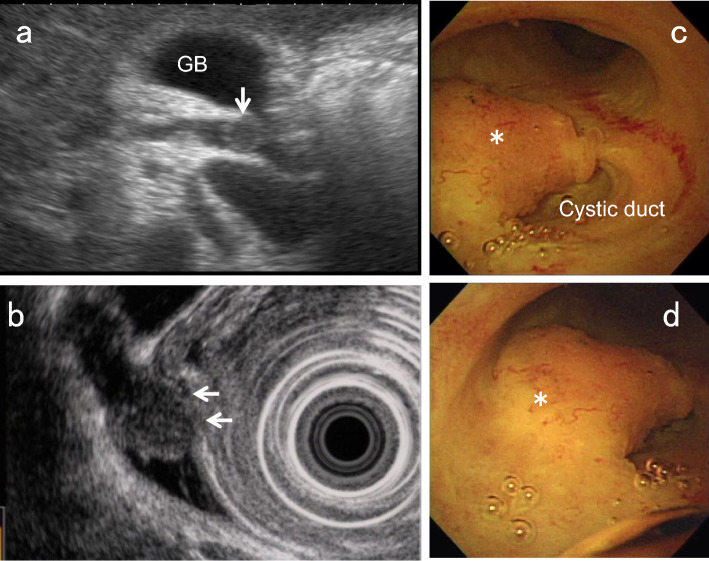
Nodular-type CCA. US shows a hypoechoic lesion with a nodular surface (↓) in the perihilar BD (**a**). EUS depicts the discontinuity of the OHL (←), suggesting tumor invasion beyond the subserosa (**b**). Peroral cholangioscopy shows a flat elevated nodular-type CCA (*) (**c**, **d**)

**Fig. 14:**
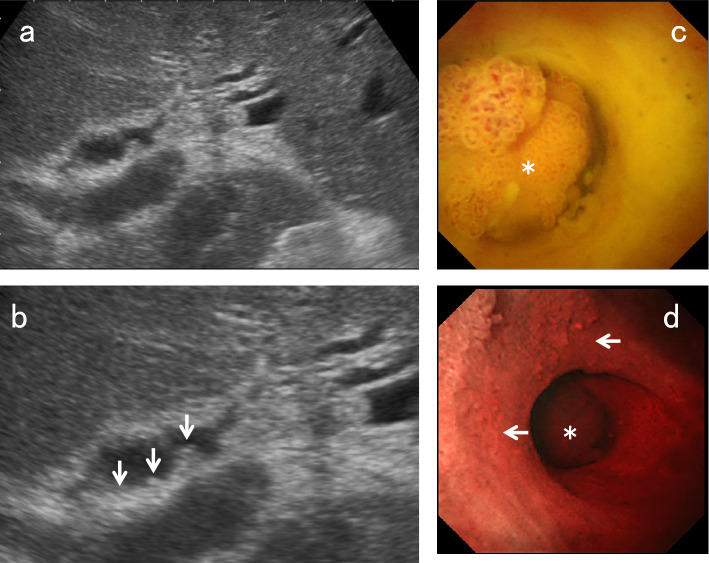
Papillary-type CCA. US shows a hyperechoic polypoid lesion in the hepatic duct (↓) (**a**) and also depicts the papillary pattern of the mucosal surface (↓), reflecting lateral tumor spreading around the main tumor (**b**). Peroral cholangioscopy shows a papillary-type CCA (*) (**c**) and its lateral spreading (←) on NBI mode (**d**)

CCA can be predominantly hypoechoic or hyperechoic or have mixed echogenicity depending on the amount of internal fibrosis, mucin, and calcification. However, nodular-type CCA tends to show heterogeneous hypoechoic to isoechoic echogenicity compared with the liver parenchyma (Fig. 13). On the other hand, papillary-type CCA (Fig. 14) and IPNB tend to show homogeneous hyperechoic to isoechoic echogenicity [18, 19], which may be due to the scattering and reflection of ultrasound.

Biliary NEN is a rare tumor that is supposed to arise from Kulchitsky cells and mostly shows heterogeneous hypoechoic echogenicity [39–42]. AN is also a rare tumor, usually arising from a remnant cystic duct after cholecystectomy, and shows homogeneous hypoechoic echogenicity [43–48]. Biliary sludge usually shows hyperechoic to isoechoic echogenicity with dotted hyperechoic spots.

### Surface contour

**Fig. 15:**
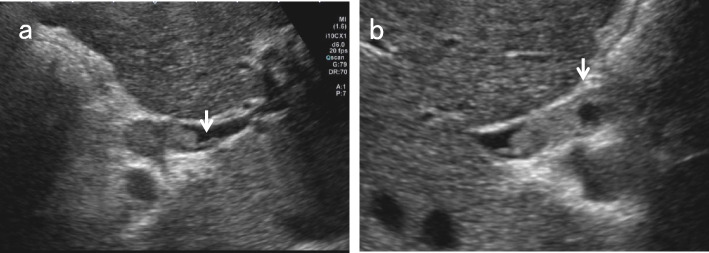
IPNB. A high-frequency transducer shows a hyperechoic to isoechoic polypoid lesion in the hepatic duct and also depicts an inner hypoechoic layer, reflecting superficial spreading (↓). (**a** Right subcostal longitudinal scan, **b** right subcostal transverse scan)

Papillary-type CCA often exhibits papillary to lobular surface structures (Fig. 14). Nodular-type CCA often presents with obstructive jaundice at the time of onset, making it difficult to evaluate the surface contour of the main lesion. However, when the surface characteristics of the hepatic extension are included, the lesion often presents as nodular with an irregular surface. On the other hand, papillary or cauliflower-shaped tumors in the expanded BD are the characteristic feature of IPNB (Fig. 15). However, it is sometimes difficult to assess the surface contour of IPNB in cases with mucus.

NEN usually shows a round shape with a smooth contour [40–42] and may resemble a submucosal tumor with a capsule [40, 42]. AN also shows a well-defined elevated submucosal tumor-like appearance with a thin normal cystic duct epithelium on cholangioscopy [46]. Both EUS and IDUS also show oval tumors with a smooth surface [43–47], and a hyperechoic rim on the surface is recognized in some cases [45, 46]. In contrast, biliary sludge is characterized by changes in shape and surface contour associated with positional changes.

### Mucosal surface around BDPLs (diagnosis of lateral spreading)

Superficial intraductal spreading is a characteristic feature of CCA [49] (Figs. 14, 15), and can be used for differential diagnosis. According to a clinicopathologic study, the frequency of superficial spreading (extending ≥ 20 mm into the mucosal epithelium) was 46.5% in the papillary type and 12.2% in the nodular type (p < 0.0001) [20].

According to an EUS study, irregularities of the IHL and/or inner hypoechoic layer reflected superficial spreading, and EUS diagnosed intraepithelial (mucosal) spreading in 52.9% of cases [50]. IDUS findings of irregularity of the mucosal surface and/or uneven or localized thickening of the IHL of the BD adjacent to the main tumor were also used in the diagnosis, and the sensitivity, specificity, and accuracy of the longitudinal extent of cancer on the hepatic and duodenal sides were 82%, 70%, and 78% and 85%, 43%, and 70%, respectively [51]. Tamada et al. reported that a papillary pattern of the BD mucosal surface and heterogeneous BD wall thickening (width 1.8 mm) with irregular outer marginal or asymmetric BD wall thickening (width 1.8 mm) with a rigid inner edge on IDUS may be signs of longitudinal spreading of CCA [52]. In studies on GB carcinoma (GBC), US, especially with a high-frequency transducer with zoom magnification, could detect slight localized thickening of the inner hypoechoic layer around GBC, which corresponds to lateral spreading of flat-type GBCs [53, 54].

Therefore, localized thickening of the internal hypoechoic layer and the papillary pattern of the mucosal surface reflect lateral tumor spreading, suggesting CCA. Since Tamada et al. emphasized the importance of asymmetrical BD wall thickening as the finding of longitudinal tumor spread [55], short-axis images of the BD are more important than long-axis images for the evaluation of lateral extension.

### Vertical layer structure of BDPLs (diagnosis of depth invasion)

As mentioned above, when employing high-frequency transducers, the BD wall structure can be identified in two or three layers: an IHL and an OHL, or an IHL, a middle hypoechoic layer, and an OHL. Here the description is divided into IHL and OHL.

#### Characteristics of the innermost hyperechoic layer (IHL)

According to studies of the GB wall, the source of the IHL is considered mostly interface echoes [22], and also include the mucosa [23]. Since BD NEN [40, 42] and AN [44, 46] are often covered by nonneoplastic mucosa and resemble a submucosal tumor, the IHL may be recognized in those cases.

#### Irregularity or discontinuity of the wall layer structure

Because tumor invasion into the adipose layer of the subserosa causes irregularity of the OHL, and tumor invasion beyond the subserosa causes disruption of the OHL, both irregularity and discontinuity of the OHL suggest wall thickening due to malignancy. However, the inner hypoechoic layer contains not only the mucosa and muscularis propria but also the fibrous layer of the subserosa [21], and an intact OHL suggests not only a benign lesion but also CCA with tumor invasion within the fibrous layer of the subserosa.

**Fig. 16:**
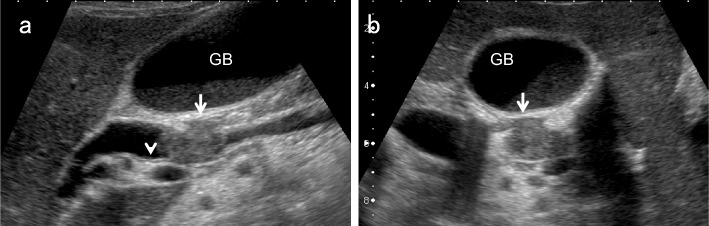
Discontinuity of OHL of nodular-type CCA. Although the long-axis image using a high-frequency transducer shows the discontinuity of the OHL (↓) and an inner hypoechoic layer, reflecting superficial spreading (**a**), the short-axis image shows the discontinuity of the OHL (↓) more clearly (**b**)

Since the muscularis propria of EHBD is thin, short-axis images with magnified images using a high-frequency transducer are strongly recommended to evaluate the irregularity or discontinuity of the wall layer (Fig. 16).

### Contrast‑enhanced US (CEUS)

According to Fontán et al. [56], CEUS demonstrated early enhancement and posterior washout in CCA. The sensitivity, specificity, positive predictive value, and negative predictive value of CEUS were 85.7%, 88.2%, 94.7%, and 71.4%, respectively. Xu et al. [57] studied the dynamic behavior of hilar CCA using CEUS, demonstrating hyperenhancement, isoenhancement, and hypoenhancement in the arterial phase in 43.8%, 43.8%, and 12.6%, respectively, and 93.8% of the cases presented washout in the portal and late phases.

The papillary or solid components of IPNB showed scarce (80%) or rich (20%) blood supply on color Doppler US [18]. On CEUS, however, they showed homogeneous hyperenhancement (92.3%) or isoenhancement (7.7%) in the arterial phase and hypoenhancement during the portal and late phases [18]. CEUS may facilitate the diagnosis of IPNB and exclude the possibility of sludge, mucus, or blood clots because they are not enhanced. AN is usually hypervascular [44–46] and uniformly enhanced at an early phase [44]. The essential contribution of CEUS at this time is differentiation among tumors and non-enhanced material, including lithiasis without acoustic shadow, blood clots, and debris [18, 36, 56]. CEUS is also useful for ruling out concomitant malignant tumors in cases with filling stone or biliary sludge.

From the above, because CCA is the most common lesion among BDPLs, it is important to diagnose tumor extension and depth invasion in addition to the differential diagnosis. Nodular-type CCA is usually hypoechoic and is more likely to invade vertically. In contrast, papillary-type CCA is often hyperechoic and tends to extend laterally. Both irregularity and discontinuity of the OHL suggest tumor invasion beyond the fibrous layer of the subserosa. Slight localized thickening of the inner hypoechoic layer and a papillary pattern on the mucosal surface around BDPLs may suggest lateral spreading of CCA.

## Conclusion

US can improve visualization of the EHBD by employing positional changes and high-frequency transducers. US can also contribute not only to the differential diagnosis of EHBD lesions but to the diagnosis of tumor extension including lateral spreading and depth invasion of CCA using magnified images with high-frequency transducers.

## Abbreviations

AN: Amputating neuroma
BDWT: Bile duct wall thickening
BDPL: Bile duct polypoid lesion
CBD: Congenital biliary dilatation
CCA: Cholangiocarcinoma
CEUS: Contrast‑enhanced ultrasound
CT: Computed tomography
EHBD: Extrahepatic bile duct
EUS: Endoscopic ultrasound
GB: Gallbladder
HRUS: High-resolution ultrasonography
IDUS: Intraductal ultrasonography
IgG4: Immunoglobulin G4
IgG4-RD: Immunoglobulin G4-related disease
IgG4-SC: Immunoglobulin G4-sclerosing cholangitis
IHL: Innermost hyperechoic layer
IPNB: Intraductal papillary neoplasm of the bile duct
MRCP: Magnetic resonance cholangiopancreatography
NEN: Neuroendocrine neoplasm
PBM: Pancreaticobiliary maljunction
OHL: Outermost hyperechoic layer
PV: Portal vein
PSC: Primary sclerosing cholangitis
US: Ultrasound
